# The leptin receptor has no role in delta-cell control of beta-cell function in the mouse

**DOI:** 10.3389/fendo.2023.1257671

**Published:** 2023-10-02

**Authors:** Jia Zhang, Kay Katada, Elham Mosleh, Andrew Yuhas, Guihong Peng, Maria L. Golson

**Affiliations:** ^1^ Department of Genetics, University of Pennsylvania, Philadephia, PA, United States; ^2^ School of Medicine, University of Pennsylvania, Philadephia, PA, United States; ^3^ Department of Medicine, Divison of Endocrinology, Diabetes, and Metabolism, Johns Hopkins University, Baltimore, MD, United States

**Keywords:** delta cells, beta-cell activity, leptin receptor (LEPR), differential expression (DE), mouse models, beta cells

## Abstract

**Introduction:**

Leptin inhibits insulin secretion from isolated islets from multiple species, but the cell type that mediates this process remains elusive. Several mouse models have been used to explore this question. Ablation of the leptin receptor (Lepr) throughout the pancreatic epithelium results in altered glucose homeostasis and ex vivo insulin secretion and Ca2+ dynamics. However, Lepr removal from neither alpha nor beta cells mimics this result. Moreover, scRNAseq data has revealed an enrichment of LEPR in human islet delta cells.

**Methods:**

We confirmed LEPR upregulation in human delta cells by performing RNAseq on fixed, sorted beta and delta cells. We then used a mouse model to test whether delta cells mediate the diminished glucose-stimulated insulin secretion in response to leptin.

**Results:**

Ablation of Lepr within mouse delta cells did not change glucose homeostasis or insulin secretion, whether mice were fed a chow or high-fat diet. We further show, using a publicly available scRNAseq dataset, that islet cells expressing Lepr lie within endothelial cell clusters.

**Conclusions:**

In mice, leptin does not influence beta-cell function through delta cells.

## Introduction

Leptin, a hormone secreted by adipose tissue, impairs hunger and promotes energy expenditure by activating its cognate receptor (1). It exerts these effects primarily through the hypothalamus (2, 3). The human and mouse leptin receptor exists in six isoforms resulting from alternative LEPR gene splicing (4). These receptors are referred to as LepRa-f. The full-length receptor, LepRb, has a strong signal in response to leptin binding. However, the short isoforms, LepRa-c and LepRf, lack varying lengths of the intracellular domain and demonstrate a weak signaling response to leptin binding (4). The short isoforms also aid in transporting leptin across the blood-brain barrier and may promote leptin internalization and degradation in some cell types (5). *Lepr^db/db^* mice become obese and, on some background strains, exhibit diabetes. *Lepr^db/db^* mice have a mutation within the *Lepr* gene that results in exon usage only for short LepR isoforms and not LepRb (6). This mutation phenocopies that of mice with a mutation in *leptin*, indicating the relative importance of the signaling capacity of the short versus long isoforms (6, 7). LepRe, known as soluble leptin receptor, is a secreted isoform lacking the transmembrane and intracellular domains. It can bind circulating leptin, thereby preventing its transport and decreasing bioavailability (8, 9).

Leptin receptor signaling acts through a variety of pathways. Leptin binding of the short or long LepR isoforms can trigger the Jak signaling cascade (4). In contrast, Signal Transducer and Activation of Transcript (STAT) protein signaling can only be activated by the full-length LepRb.

In addition to its hypothalamic localization, the leptin receptor (LEPR) is also expressed in immune cells, pericytes, and endothelial cells, affecting various immune processes and vessel constriction (4, 10). LepRb has also been detected by RT-PCR and Northern blot in islets, and multiple labs have reported that leptin directly inhibits insulin secretion from isolated mouse, human, and rat islets (11–17). Moreover, deletion of the leptin receptor (*Lepr*) throughout the pancreatic epithelium using *Pdx1*-*Cre* alters glucose homeostasis (18). *Pdx1-Cre;Lepr^fl/fl^* mice fed a normal chow diet display improved glucose tolerance, while those provided a high-fat diet display glucose intolerance (18). Since leptin inhibits insulin secretion, deleting *Lepr* would be expected to promote insulin secretion, as observed on a normal chow diet. Perhaps this increased insulin secretion in times of high metabolic demand, such as when on a high-fat diet, leads to beta-cell exhaustion and thus the glucose tolerance observed in high-fat-diet-fed *Pdx1-Cre;Lepr^fl/fl^* mice.

While the altered glucose homeostasis in *Pdx1-Cre;Lepr^fl/f^* suggests that the cell responsible for this phenotype has an epithelial origin, narrowing down the exact cell type has proven difficult. Two different models of *Lepr* deletion within beta cells have yielded conflicting results. *Rat insulin promoter* (*RIP^25Mgn^)-Cre;Lepr* mice display hyperglycemia and reduced insulin secretion, but they also have high adiposity due to RIP-Cre^Mgn^ activity in the brain (19, 20); the disrupted glucose homeostasis is likely a secondary effect to obesity associated with loss of Lepr in the hypothalamus. A second model of beta-cell *Lepr* ablation using *Insulin 1 (Ins1)-Cre* results in only a slight change in blood glucose regulation, with females at eight weeks of age exhibiting slightly improved glucose tolerance and enhanced glucose-stimulated insulin secretion (21). These changes disappeared by sixteen weeks of age. In addition, alpha-cell ablation of *Lepr* using either *Glucagon* (*Gcg*)*-Cre* or *Gcg-Cre^ER^* did not lead to altered glycemia (21, 22).

**Figure 1:**
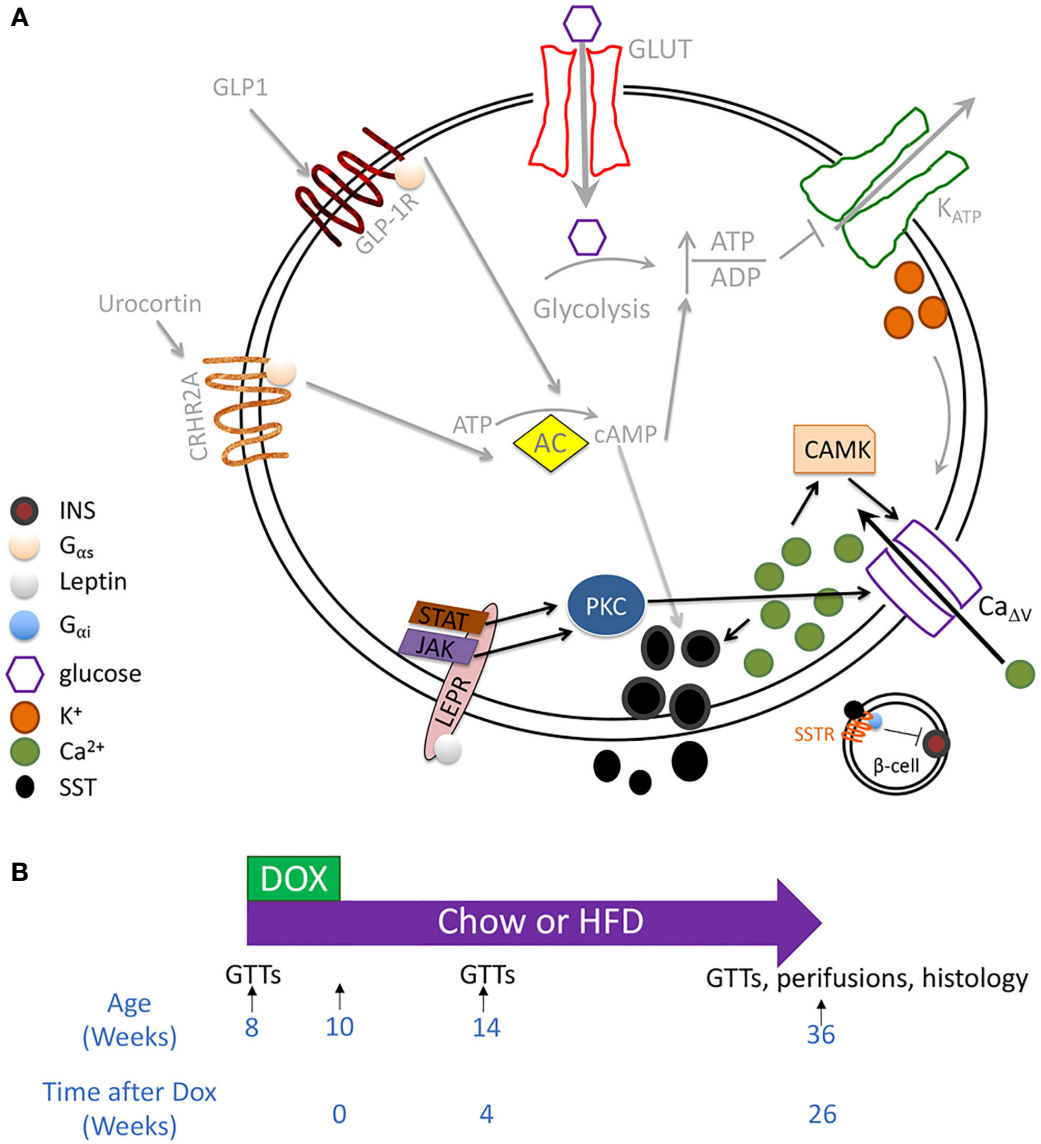
Testing whether leptin mediates inhibition of insulin secretion through the delta cell. **(A)** RNAseq of sorted mouse alpha, beta, and delta cells has revealed the expression of all the components involved in canonical and amplifying pathways of insulin secretion, except insulin itself, in the delta cell. These components include a glucose transporter, voltage-gated potassium channels, glucokinase, and adenylate cyclase. Delta cells are also regulated by paracrine interactions by other endocrine cells, including by Urocortin 3 and by GLP1 (30–34). Although cytosolic Ca^2+^ may be secondary to cAMP in regulating delta-cell function, Ca^2+^ concentration correlates with somatostatin secretion. In human islets, the leptin receptor is enriched within the delta cell population. We, therefore, hypothesized that leptin receptor signaling enhances SST release by activating a PKC isoform that enhances Ca_Δv_ activity and increases intracellular Ca^2+^ (pathway denoted by black arrows). Proposed leptin receptor signaling is shown with black arrows while established signaling pathways in the delta cell are depicted with grey arrows. **(B)** Mouse model: *Sst^rtTA^*;*Tet-O-Cre;Lepr^fl/fl^* (*Lepr^Δδ^*) and littermate control mice were placed on doxycycline from eight weeks to ten weeks of age. Mice were assigned to a high-fat or normal chow diet at ten weeks of age. GTTs were performed at eight, 14, and 36 weeks of age, corresponding to before dox treatment, and four and 26 weeks after doxycycline cessation.

Another primary islet endocrine cell type is the somatostatin-secreting delta cell. Somatostatin is a paracrine hormone that inhibits adjacent secreting cells. Single-cell RNA sequencing (scRNAseq) of human islets has revealed that *LEPR* is enriched within delta cells (23). Leptin signaling is generally inhibitory for secretory cells since the Jak cascade through phosphoinositide 3 kinase and phosphodiesterase 3B ultimately converts cAMP into AMP (24). However, LepR signaling through Jak or STAT can activate PKC (25, 26). In multiple cell types, including beta cells and heart cells, some PKC isoforms enhance Ca^2+^ influx and insulin secretion (27, 28). Delta cells are glucose-responsive (29). In addition to expressing all the machinery for insulin secretion, except insulin itself, they also integrate several paracrine signals, such as GLP-1 and Urocortin 3, which elevate cAMP levels, and electrical signals from beta-cell through gap junctions (16, 30–33) (**Figure 1A**). Although cAMP likely has a more prominent role in regulating delta-cell than beta-cell function, delta-cell Ca^2+^ concentration is correlated with somatostatin secretion (29, 35). Considering these data, we proposed that leptin acts on delta cells by stimulating PKC, elevating Ca^2+^ and somatostatin release, thereby inhibiting beta cells (**Figure 1A**). We tested this premise in a mouse model of delta-cell-specific *Lepr* ablation.

## Materials and methods

### Mice

*Sst-rtTA* (36), *Tet-O-Cre (* 37 *)*, and *Lepr^fl/fl^* (38) mice and genotyping have been described previously. 2% doxycycline in water was administered at eight weeks of age for two weeks. Mice were fed chow or 60% HFD (Research Diets) *ad libitum* and maintained on a 12-hour light/dark cycle. Control mice were littermates of mutants, had one allele of *Sst^rtTA^*, and included *Sst^rtTA^;Tet-O-Cre;Lepr^+/fl^* and *Sst^rtTA^;Lepr^fl/fl^* mice. All procedures followed the Institutional Animal Care and Use Committee guidelines at the University of Pennsylvania or Johns Hopkins University.

### Glucose tolerance tests, mouse islet isolations, and perifusions

For glucose tolerance tests (GTTs), mice were fasted overnight for 16 hours, and blood glucose was measured with an AlphaTRAK glucometer. Mice were injected intraperitoneally with 1g/kg or 2g/kg glucose in sterile 1X PBS; blood glucose was measured 15, 30, 60, 90, and 120 minutes after injection. Islets were isolated by collagenase digestion followed by Ficoll separation and hand-picking, as previously described (39). Perifusions were performed by the University of Pennsylvania Pancreatic Islet Cell Biology Core.

### Immunofluorescence and immunohistochemistry

Pancreata were fixed in 4% PFA for 4 hours, rinsed in 1X PBS, and embedded in paraffin. 4-micron sections were attached to PermaFrostPlus slides. For beta-cell mass assessments, five sections spread throughout the pancreas were immunolabeled with guinea pig-anti-insulin (DAKO) and a secondary biotinylated anti-guinea pig antibody. HRP was bound to the antibody with an ABC kit (Vector Laboratories) and visualized with DAB (Vector Laboratories). Slides were counterstained with eosin. Imaging was performed with an Aperio microscope at 400X. DAB- and eosin-positive areas were quantified using QuPath (40). The DAB-to-eosin ratio was multiplied by pancreas weight to obtain beta-cell mass. For immunofluorescence, the primary antibodies guinea pig-anti-insulin (1:500, DAKO), mouse anti-SST (1:250, Santa Cruz), and rabbit anti-glucagon (1:500, Santa Cruz) were used. Secondary antibodies (Jackson Immunolaboratories) were applied at 1:600. Nuclei were stained with DAPI. Images were acquired using a BZ-X Series All-in-One Fluorescence Microscope.

### Recombination analysis

One to four paraffin-embedded pancreatic sections were scraped with a clean razor into a sterile microcentrifuge tube and digested overnight with 100 microliters of Proteinase K solution before standard PCR, using 1-10 microliters of heat-inactivated digested sections. Primers for recombination analysis were previously described (41).

### qPCR

Primers (*Lepr* F 5’-GTT TCA CCA AAG ATG CTA TCG AC-3’; *Lepr* R 5’GAG CAG TAG GAC ACA AGA GG-3’) for *Lepr* were tested on hypothalamus cDNA before analyzing *Lepr* expression in islets. These primers span exons 18-19 and demonstrated the same melting curve product in islet and hypothalamic cDNA. RNA was isolated and cDNA was generated from whole islets or hypothalamus using Trizol plus the Qiagen RNeasy kit, as previously described (42). cDNA was synthesized using 100 ng RNA, as previously described (42). qPCR was performed using 1/3 of the total cDNA made, spread over six wells (42).

### Bulk RNAseq

Human islets were obtained from the Integrated Islet Distribution Program (IIDP). Islet preparation and RNA extraction after intracellular marker cell sorting have been described previously (43). Briefly, islets from five non-diabetic human donors (**Table 1**) were suspended into a single-cell solution using 0.05% Trypsin, then fixed in 1% PFA, 0.1% saponin, and 1:100 RNasin (Promega). Cells were then indirectly fluorescently immunolabeled against somatostatin and insulin before sorting. RNA was extracted with a Recoverall Total Nucleic Acid kit (Life Technologies). RNA library was prepared and sequenced as previously described (44). Data was analyzed by the University of Pennsylvania Next Generation Sequencing Core, as previously described (44).

**Table 1:** Donor characteristics for bulk RNAseq.

Donor	Sex	Age	BMI	Race/Ethnicity
1	M	35	23.6	White
2	F	53	20.5	White
3	F	36	35.7	Hispanic
4	F	24	35.3	White
5	M	25	24.3	Black

### scRNAseq

scRNAseq data was downloaded from PANC-DB (pmacs.hpap.upenn.edu) on March 31, 2023, using cell calling performed by the Human Pancreas Analysis Program (HPAP). Non-diabetic, autoantibody-negative male and female donors >17 years of age were included in our analysis. Differential expression analysis was performed as previously described (39).

## Results

### Differential expression between bulk-sorted human beta and delta cells

Current sorting protocols for live human islet cells cannot reliably distinguish delta cells from beta cells (45, 46). Therefore, gene expression differences have been reported between bulk-sorted human alpha and beta cells (47) but not between bulk-sorted human beta and delta cells. To separate these two closely related cell types, we fixed islets from five non-diabetic human donors, suspended them in a single-cell solution, permeabilized them, and indirectly immunolabeled them with antibodies against insulin and somatostatin. These cells then underwent fluorescence-activated cell sorting. RNA was extracted from the purified beta- and delta-cell populations and subjected to RNAseq and analysis. As expected, known lineage-specific genes such as *SST* and *HHEX* were enriched in delta cells (**Table 2**), while genes such as *INS* and *MEG3* were more highly expressed in beta cells (**Table 3**).

**Table 2:** Top enriched genes in sorted delta cells compared to sorted beta cells.

Gene Symbol	Fold Enrichment	FDR
SST ERBB4 SLITRK6 SALL1 AGTR1 MS4A8B HHEX KCNIP1 CBLN4 EPHA6 TMEM132E ODZ2 IGSF10 DCHS2 FLRT1 DRD2 GABRA1 PDE2A SLC5A8 LRFN5 NTNG1 FAM5C LRRC4C BCHE LPHN2 C1orf173 KCNT2 FHOD3 SRRM4 PREX2 PPFIA2 LEPR RYR1 GABRA5 KCNC2 LOC100233209 ODZ1 SORCS1 LEPR PDE1C FAM113B ARHGEF38 CPA4 SEMA3E MLPH SERPINA1 RGS6 GABRG2 LAMA1	283.354336 231.194075 182.211784 173.469728 126.263771 121.274614 115.78778 99.9023306 98.1777133 92.2108402 86.6689936 74.9405509 71.1360064 70.6242855 70.407892 70.2015891 67.364591 64.9836722 64.1198737 63.8498264 62.2271069 60.5572346 55.5375203 55.1893356 53.0600885 52.1434247 50.3648355 48.5091315 47.7582127 46.0390162 44.9601447 44.844213 44.183812 43.5690639 40.9824238 40.6414705 40.6015994 39.5170708 39.2396012 38.7210966 38.5093709 35.7514589 35.7382946 35.5044204 35.392378 35.0834445 34.7928073 34.2872608 32.8074723	3.2099E-145 1.56518E-71 4.29086E-57 3.27556E-71 3.30735E-43 4.99604E-70 1.50192E-81 7.06592E-51 4.44692E-39 8.98006E-33 3.49776E-31 1.00547E-60 2.32374E-60 1.42405E-62 9.4954E-52 5.55123E-33 2.03333E-26 4.78243E-23 4.02285E-26 9.00996E-94 1.84028E-46 1.44708E-16 4.22091E-18 3.90434E-54 4.78117E-82 5.11576E-65 8.46998E-27 6.404E-65 4.48161E-23 1.45321E-27 1.52849E-69 2.30687E-97 3.95159E-29 7.12644E-21 1.66405E-27 1.11879E-21 1.99714E-29 8.74982E-16 2.91084E-77 4.91781E-23 2.17406E-27 1.99714E-29 5.60967E-32 4.55075E-49 6.38692E-43 3.17001E-26 1.3526E-28 2.23566E-10 3.07579E-19

**Table 3:** Top enriched genes in sorted beta cells compared to sorted delta cells.

Gene Symbol	Fold Enrichment	FDR
PPM1E PCA3 ZNF114 SNORD113-1 PRKCH ZPLD1 TRIM9 TSPAN1 MEG8 MEG3 IGSF11 WSCD2 IGFBP3 SNORD114-31 TDRG1 TMEM108 TFF3 LOC285501 LY75 OTOGL PTGS2 TNFRSF11A ROBO2 LPAR4 SLC2A2 PPP2R2C HOPX ZNF385D SNORD114-12 NCAN MFI2 TCERG1L SNORD114-11 SNORD114-7 KCNG3 SLC6A6 LRFN2 FOXQ1 SNORD114-2 HAPLN4 INS-IGF2 SPAG6 RPH3A TM6SF2 LOC145837 HS6ST2 RGS16 TNNI1 MAF HHATL INS	77.3401176 60.7783158 56.2000104 56.1416837 54.2072419 53.8064123 51.9466875 49.7902167 44.8834693 41.8423225 41.7490255 38.6772911 37.994922 37.0304821 35.9852021 35.5838299 35.1481675 34.4432135 31.8524874 31.2755596 30.9494755 30.8657815 29.024644 28.8794137 27.9735477 27.0457367 26.8617054 26.5909198 24.9059593 23.9614853 23.8734734 23.8421319 23.230222 23.2297229 22.0542854 21.9826146 21.4123197 21.250562 20.5709127 20.5445129 20.3647453 19.6697994 19.5420834 19.5391075 19.5333186 19.2960624 19.0761522 19.0404075 18.7190384 18.698355 18.6035554	1.44869E-38 6.30411E-32 2.03476E-27 2.3718E-26 4.53726E-36 6.9859E-25 2.03706E-29 1.58607E-52 1.5268E-26 1.90814E-21 8.9035E-13 6.7188E-50 1.71278E-19 1.25214E-48 1.89274E-66 4.82458E-41 4.49843E-92 5.43917E-15 3.51209E-24 4.12386E-13 6.76815E-28 2.33819E-50 1.3592E-23 1.96509E-26 2.91653E-20 2.13994E-19 1.45504E-17 2.98103E-21 2.65463E-36 1.52079E-20 1.13795E-21 1.22641E-20 1.79034E-27 8.24218E-45 4.43018E-08 8.06183E-43 1.34912E-15 4.17158E-08 2.10688E-05 1.13442E-11 9.97745E-10 1.26075E-30 4.53605E-10 1.06378E-26 4.58414E-11 1.51035E-09 4.93904E-27 1.84728E-37 1.24332E-14 5.98751E-08 1.01903E-09

An additional gene enriched within delta cells was *LEPR*, in agreement with a previous scRNAseq study (23). *LEPR* appears twice, once as the full-length (NM_002303, >39-fold enriched) and once as a short (NM_001198689, >44-fold enriched) isotype. Since our bulk-sorted RNAseq was performed on a mixture of male and female donors, we also examined gene expression differences between male and female beta and delta cells using publicly available scRNAseq data from the Human Islet Research Network’s Human Pancreas Analysis Program (**Table 4**; (48)). The genes enriched within male and female delta cells were similar, although the magnitude of differential expression varied. *LEPR* was elevated in delta cells compared to beta cells by 1.490-fold and 1.328-fold with an adjusted p-value of 3.80 x 10^-308^ or 9.99 x10^-311^ in females and males, respectively. Although *LEPR* was not among the top 50 enriched delta-cell genes in males, it was in females (**Table 4**). The difference in enrichment of *LEPR* in human scRNAseq data and bulk-sorted data was observed in most genes and likely reflects the drop-outs that occur in scRNAseq, since only a fraction of expressed genes are detected in any given cell, thus lowering mean expression for the population (49).

**Table 4:** Top delta-cell enriched genes compared to beta cells from human (HPAP) scRNAseq samples ranked firstly by female fold enrichment and secondly by male fold enrichment.

Gene symbol	Female fold enrichment	Female adjusted p-value	Gene symbol	Male Fold Enrichment	Male Adjusted p-value
SST RBP4 SERPINA1 LY6H HHEX CLU MDK FAM81B RGS2 S100A10 LEPR TM4SF4 S100A6 PDLIM4 SLC38A1 PRG4 LINC01571 TMEM176A NCOA7 FXYD6 F5 LDHA TMSB4X TMEM176B SPTBN1 ISL1 BCHE UNC5B USH1C DIRAS3 AKAP12 AQP3 CBLN4 SSTR1 S100B TJP2 RHOC TFPI SMIM24 HLA-B SPATS2L CRIM1-DT FABP5 GSTP1 NNMT POU3F1 FRZB GPC5-AS1 FXYD3 SEMA3E SSX2IP CALY* SEC11C* PLD3* CD9* H3F3B PCSK1N^ζ^ HMGN2* MTRNR2L12^ζ^ ARFGEF3* IDS^ζ^ HLA-A^ζ^ CHGB GPX4 C9orf16^ζ^ EMC10 RBP1 PCSK2 BEX3 BTG2 B2M PEG10 TIMP1^ζ^ UQCR10^ζ^ SELENOM^ζ^ GABRB3 H3F3A^ζ^ MAP1B EPCAM^ζ^ EIF5 COX8A^ζ^ ITM2C GPX3^ζ^ CETN2	99.7510623 4.56649388 3.68034293 2.43289575 2.12443691 1.89978084 1.70683417 1.70621742 1.59736289 1.516391 1.49506665 1.43562196 1.4125767 1.41218304 1.38512195 1.37369412 1.36560836 1.36556426 1.364086 1.35542774 1.34688194 1.33069054 1.3147048 1.30105874 1.29849396 1.2829392 1.28257296 1.27693572 1.26345878 1.25512991 1.23511339 1.23480206 1.23327625 1.2248029 1.22179378 1.21182124 1.21036351 1.20503538 1.19805098 1.19438413 1.18721078 1.18568419 1.18005475 1.17726638 1.17514054 1.17511072 1.17391553 1.1721296 1.1699364 1.16451393 1.16364363 1.07828923 0.76153899 1.06291115 1.15124644 No difference 0.59920831 No difference 0.680871 1.11729566 0.8549307 0.88435405 No difference 0.83691184 0.79750944 1.03008708 No difference No difference No difference No difference No difference No difference 0.74074691 0.82987633 0.77018889 1.15553583 0.76387302 No difference 0.72465184 No difference 0.79931031 No difference 0.81443445 No difference	3.80E-308 3.80E-308 3.80E-308 3.80E-308 3.80E-308 3.90E-177 9.28E-168 3.80E-308 3.25E-145 6.41E-129 3.80E-308 1.47E-42 1.50E-58 2.04E-180 3.80E-308 3.00E-195 3.80E-308 4.37E-87 4.12E-132 3.63E-104 3.80E-308 1.03E-162 4.84E-11 1.47E-49 1.45E-66 9.56E-14 3.80E-308 3.97E-87 2.15E-106 6.01E-98 6.67E-125 2.02E-05 3.80E-308 3.69E-128 4.32E-138 1.30E-223 1.81E-155 9.80E-237 7.79E-42 2.79E-06 5.94E-70 1.74E-82 1.33E-15 2.73E-32 9.33E-80 9.46E-122 2.08E-151 2.30E-282 9.55E-35 5.87E-152 3.84E-28 8.22E-21 8.57E-16 4.07E-11 6.49E-35 3.10E-73 1.03E-53 6.67E-10 4.23E-06 0.88435405 3.26E-23 6.64E-27 9.92E-06 2.09E-23 2.90E-13 2.44E-08 3.99E-21 1.22E-32 1.24E-36 3.50E-26 7.73E-06	SST RBP4 SERPINA1 LY6H HHEX CLU* MDK FAM81B* RGS2 S100A10 LEPR* TM4SF4* S100A6* PDLIM4* SLC38A1* PRG4* LINC01571* TMEM176A* NCOA7* FXYD6* F5* LDHA* TMSB4X* TMEM176B SPTBN1* ISL1* BCHE* UNC5B* USH1C* DIRAS3* AKAP12* AQP3* CBLN4* SSTR1* S100B* TJP2* RHOC* TFPI* SMIM24* HLA-B* SPATS2L* CRIM1-DT FABP5* GSTP1* NNMT* POU3F1* FRZB* GPC5-AS1* FXYD3* SEMA3E* SSX2IP* CALY SEC11C PLD3 CD9 H3F3B PCSK1N HMGN2 MTRNR2L12 ARFGEF3 IDS HLA-A CHGB GPX4 C9orf16 EMC10 RBP1 PCSK2 BEX3 BTG2 B2M PEG10 TIMP1 UQCR10 SELENOM GABRB3 H3F3A MAP1B EPCAM EIF5 COX8A ITM2C GPX3 CETN2	74.064405 5.83781281 2.30593345 2.00118119 1.74480337 2.54570509 1.73497984 1.4033809 1.61626575 1.58718966 1.32781259 1.22421857 1.79493681 1.42330649 1.26061123 1.47664258 1.37907485 1.4260657 1.39456703 1.37937518 1.26854739 1.22603071 No difference 1.51253538 1.26875831 1.49692844 1.2702367 1.36978984 1.25512606 1.31984539 1.30470169 1.18980266 1.21332645 1.15786118 1.22003811 1.14752048 1.11891557 1.08644735 No difference No difference 1.17838943 1.18276205 No difference 1.6478064 No difference No difference No difference No difference 1.17548022 1.13887333 1.2915782 1.75407152 1.72844554 1.69368448 1.68865309 1.6396624 1.59358038 1.58211353 1.55021274 1.54743013 1.54405585 1.53396326 1.51273068 1.49824245 1.48684411 1.46451592 1.46367132 1.4561786 1.45417277 1.45040665 1.44664736 1.44336986 1.43581254 1.43030968 1.42824642 1.4233065 1.42034585 1.41856548 1.41535273 1.40662724 1.40620788 1.40297062 1.39759199 1.39491316	9.28E-308 5.14E-234 6.48E-116 9.28E-308 9.28E-308 2.74E-73 7.21E-109 3.56E-157 5.32E-109 1.48E-37 9.28E-308 6.80E-62 1.43E-47 1.27E-46 1.56E-127 1.59E-97 9.28E-308 9.22E-59 6.89E-87 6.50E-101 1.12E-200 1.67E-56 1.62E-60 4.09E-19 1.73E-59 4.80E-238 5.17E-129 3.89E-73 1.97E-86 3.93E-82 1.09E-20 1.93E-242 2.89E-73 3.27E-100 3.29E-53 6.43E-30 1.05E-11 6.24E-21 9.01E-67 4.33E-37 3.80E-13 1.93E-58 4.15E-39 1.23E-43 5.73E-23 5.43E-47 1.47E-78 9.03E-25 7.76E-19 6.58E-21 2.42E-10 1.79E-40 6.75E-21 1.55E-07 2.35E-10 2.71E-11 5.65E-16 8.48E-35 3.69E-25 3.97E-26 5.04E-23 3.19E-27 9.35E-07 5.56E-16 3.77E-14 8.45E-19 6.20E-24 1.27E-46 9.82E-11 1.39E-21 2.96E-13 1.25E-25 1.24E-14 3.54E-27 4.81E-25 2.27E-25

Asterisks denote genes with differing rank order. “No difference” denotes no differential expression compared to beta cells. ^ζ^ denotes opposite directions of differential gene expression.

### Delta-cell-specific deletion of Lepr in mice

Ablation of the *Lepr* within the mouse pancreatic epithelium results in alterations in glucose homeostasis, whereas ablation within alpha or beta cells does not. Together with the enrichment of *LEPR* in human delta cells, these results suggested to us that delta cells mediate the inhibited insulin secretion observed in response to leptin treatment. We, therefore, derived a mouse model (*SST^rtTA/+^;Tet-O-Cre;Lepr^fl/fl^*, henceforth referred to as *Lepr^Δδ^* mice) to test this idea (**Figure 1B**). Mice were treated with doxycycline for two weeks. In addition to its presence in islet delta cells, somatostatin is also expressed in gut enteroendocrine cells. We, therefore, performed a four-week washout to allow the replenishment of intestinal SST^+^ cells without *Lepr* ablation before performing any phenotypical examinations. While the regeneration of islet delta cells from an unrecombined progenitor population rarely occurs, intestinal D cells regenerate within two weeks, while gastric D cells demonstrate apparent regrowth within four weeks and completely recover within three months (50).

### Analysis of Lepr gene recombination and RNA expression

**Figure 2:**
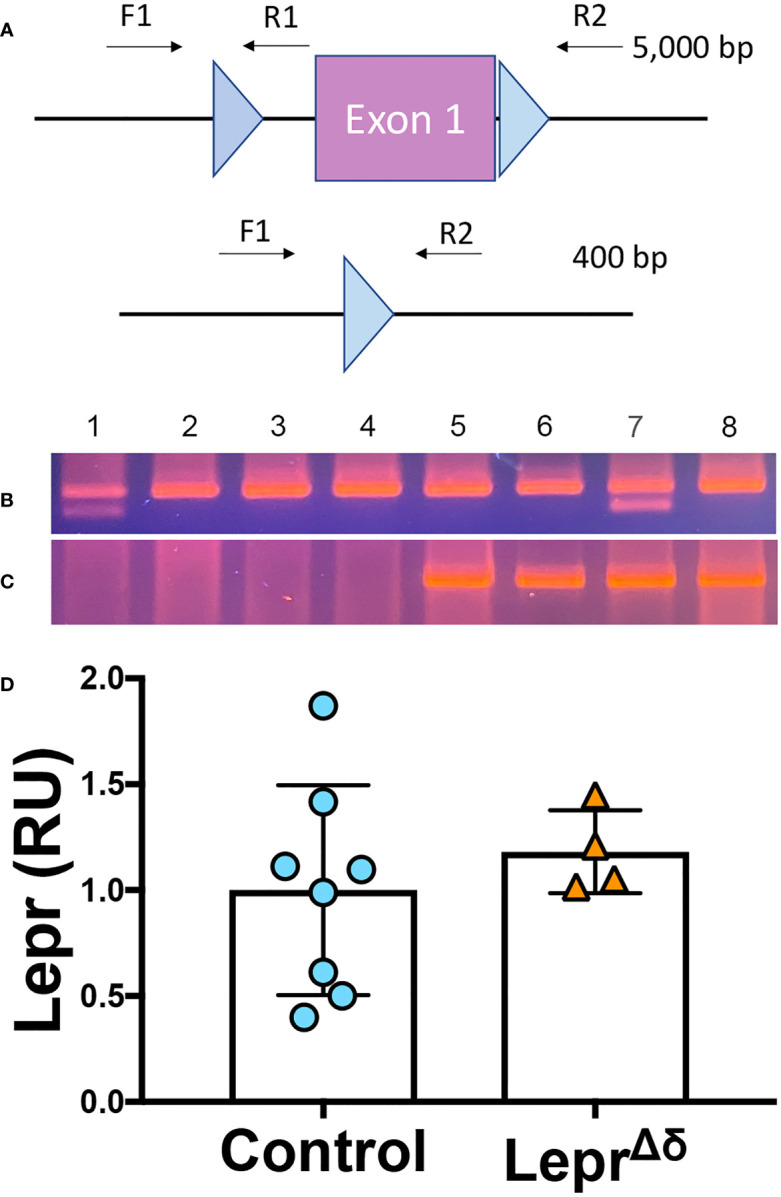
*Lepr* recombination and expression analysis. **(A)** Schematic of *Lepr^fl^* allele and primers used to assess DNA recombination of the loxP elements. **(B, C)** PCR was performed on DNA collected from fixed pancreatic sections. F1/R1 primers amplify unrecombined DNA **(B)**, while F1/R2 primers result in a ~400bp product after *Lepr^fl^* recombination **(C)**. All samples tested had one allele of *Sst^rtTA^.* Samples 1 and 7 were heterozygous for *Lepr^fl^*, and Samples 2,3,4,5,6, and 8 were homozygous for *Lepr^fl^*. Samples 1-4 were *Tet-O-Cre-*negative, while Samples 5-8 were *Tet-O-Cre-*positive. D) qPCR analysis of *Lepr* expression in whole islets from *Lepr^Δδ^* or control mice.

We next examined *Lepr* locus recombination using DNA extracted from pancreatic sections from 36-week-old *Sst^rtTA/+^;Tet-O-Cre;Lepr^+/fl^*, *Sst^rtTA/+^;Tet-O-Cre;Lepr^fl/fl^*, *Sst^rtTA/+^;Lepr^+/fl^*, and *Sst^rtTA/+^;Lepr^fl/fl^* mice treated with doxycycline from eight to ten weeks of age. The *Lepr^fl^* allele was designed with loxP elements flanking the first exon of *Lepr* (38). We used primer sets spanning either one loxP element or both loxP elements to directly examine DNA recombination (**Figure 2A**). Primers traversing only one loxP element amplify unrecombined DNA, which in our model would be expected from non-delta cells. Amplification with these primers resulted in one (*Lepr^fl/fl^* mice) or two bands (*Lepr^+/fl^*) in all samples (**Figure 2B**), with one band indicating homozygosity of the floxed allele and two bands indicating one wild-type and one floxed allele. The high intensity of these bands remaining in Cre-positive animals reflects the large number of non-delta cells in the pancreas.

In the absence of recombination, primers flanking both loxP elements span an approximately 5kb distance, a distance too long for amplification using standard PCR reagents (**Figure 2A**). A recombined *Lepr* gene would result in an approximately 400-bp amplicon. In the absence of *Tet-O-Cre*, no band was observed. All samples from mice harboring at least one allele of *Sst^rtTA^, Tet-O-Cre*, and *Lepr^fl^* yielded the 400-bp band expected when recombination occurs (**Figure 2C**), likely reflecting DNA alterations in the delta cells.

To test whether *Lepr* expression was reduced in *Lepr^Δδ^* delta cells, whole islets were isolated from 14-week-old *Lepr^Δδ^* and control mice for quantitative RT-PCR analysis. No difference was observed in *Lepr* expression between *Lepr^Δδ^* and control mice (**Figure 2D**). Others have reported decreased *Lepr* expression when this *Lepr^fl^* mouse was used for deletion in other tissues (38). No difference in mRNA, together with the recombination observed in some pancreatic cells, suggested that *Lepr* is expressed in cells other than delta cells within the islet. We reasoned that we were not detecting *Lepr* deletion because delta cells make up only a small percentage of mouse islet endocrine cells (~5%), and expression in other cell types would overwhelm differences generated by *Lepr* absence in delta cells.

### Delta-cell-specific Lepr ablation in the mouse results in normal glucose homeostasis and insulin secretion

**Figure 3:**
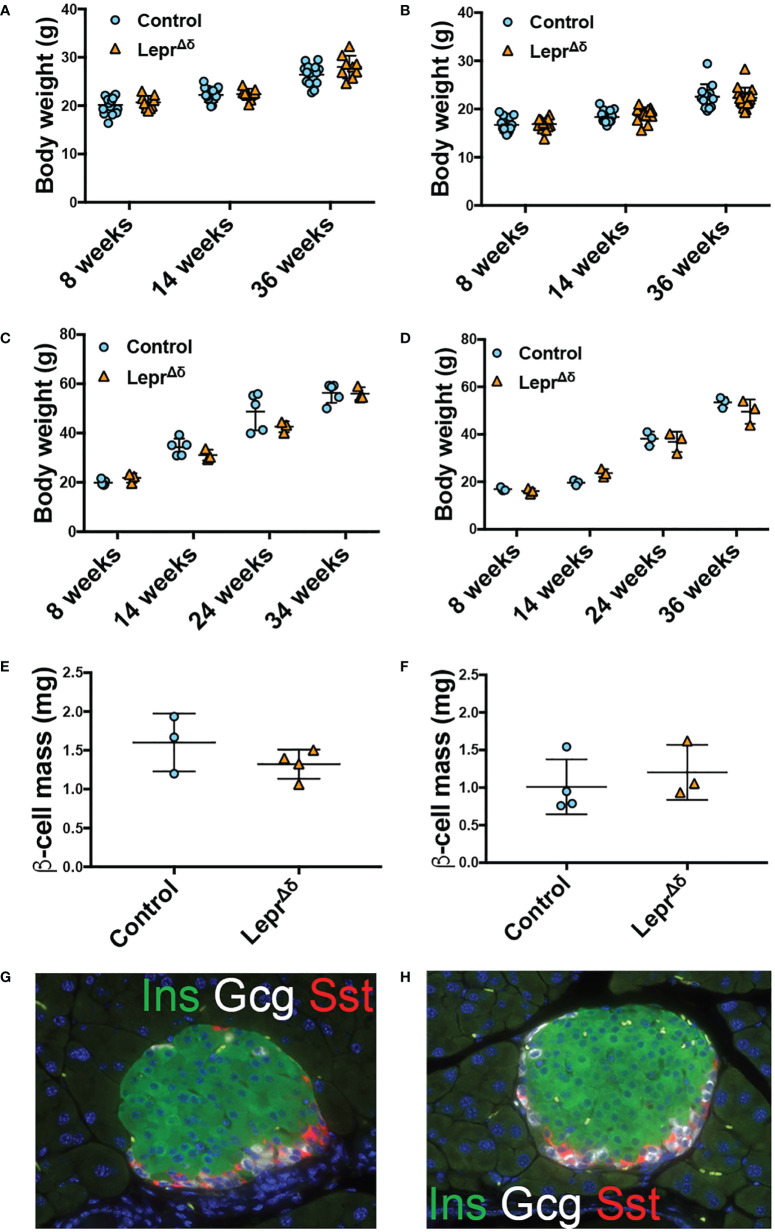
*Lepr^Δδ^* mice display unaltered weight, beta-cell mass, and islet morphology. **(A, B)** Body weight for **(A)** males and **(B)** females on chow diet. **(C, D)** Body weight for **(C)** males and **(D)** females on high-fat diet. E-F) Beta-cell mass at 36 weeks of age for males **(E)** and females **(F)** on HFD. **(G, H)** Representative image of islets from control **(G)** and *Lepr^Δδ^* **(H)** mice taken at 400X.

No difference in weight was observed between *Lepr^Δδ^* and control mice on a high-fat or chow diet (**Figures 3A-D**). There were no significant differences in beta-cell mass (**Figures 3E, F**) or islet morphology (**Figures 3G, H**).

**Figure 4:**
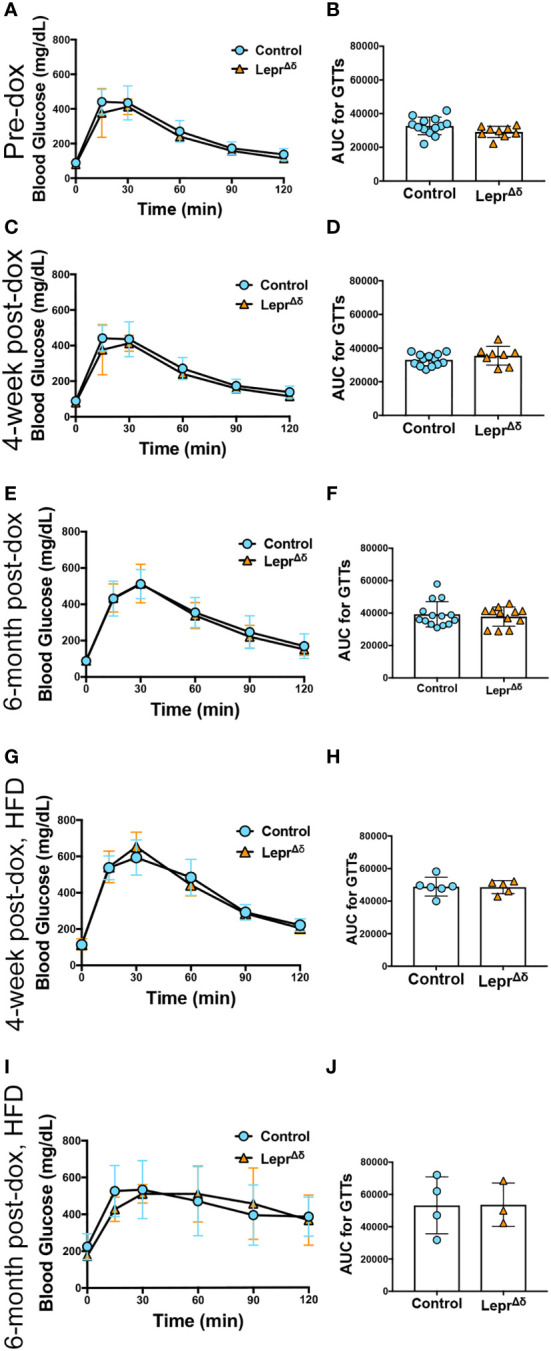
*Lepr^Δδ^* male mice display normal glucose tolerance on chow and high-fat diet. **(A-J)** GTTs **(A, C, E, G, I)** and corresponding area-under-the-curve analysis **(B, D, F, H, J)** for male *Lepr^Δδ^* mice prior to doxycycline administration **(A, B)**, at 14 weeks of age and four weeks after doxycycline removal and placement on a chow diet **(C, D)**, at 36 weeks and six months after doxycycline removal and placement on a chow diet **(E, F)**, at 14 weeks of age and four weeks after doxycycline removal and placement on a high-fat diet **(G, H)**, and at 36 weeks of age and 26 weeks (6 months) after doxycycline removal and placement on a high-fat diet.

**Figure 5:**
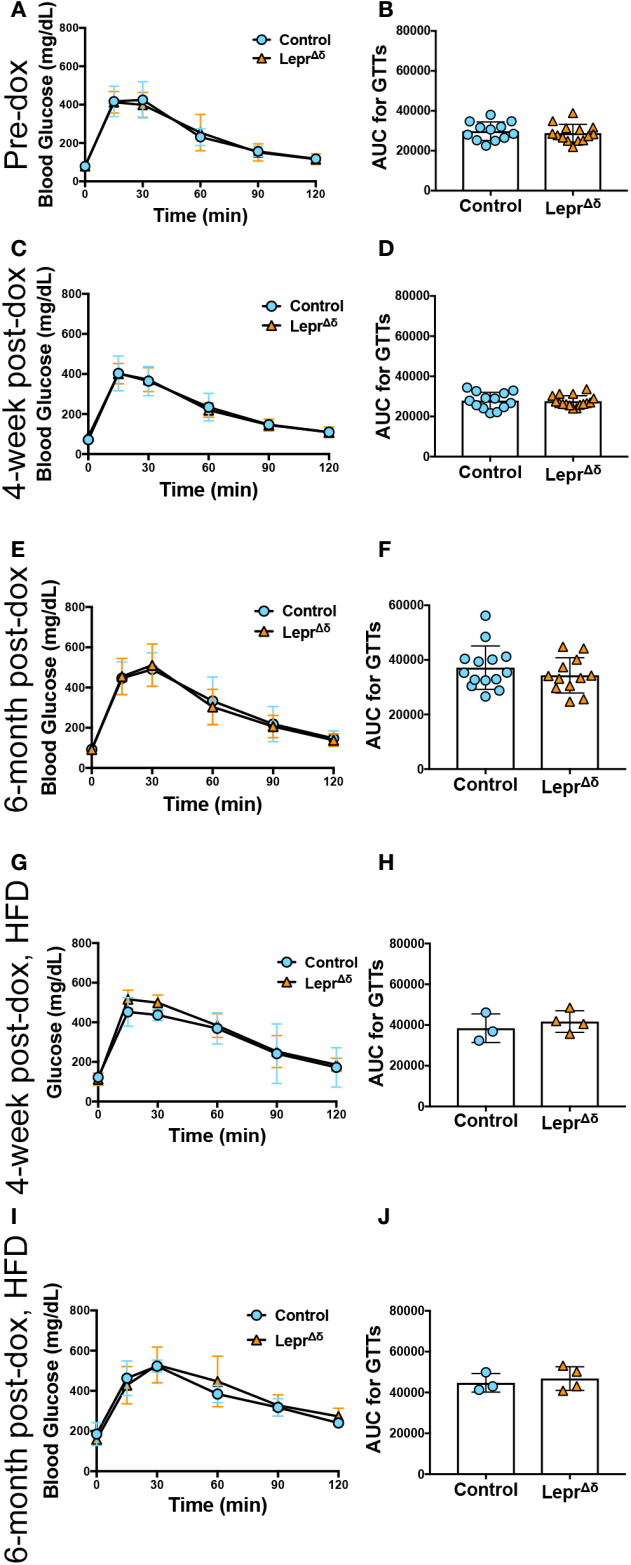
*Lepr^Δδ^* female mice display normal glucose tolerance on chow and high-fat diet. **(A-J)** GTTs **(A, C, E, G, I)** and corresponding area-under-the-curve analysis **(B, D, F, H, J)** for female *Lepr^Δδ^* mice prior to doxycycline administration **(A, B)**, at 14 weeks of age and four weeks after doxycycline removal and placement on a chow diet **(C, D)**, at 36 weeks and six months after doxycycline removal and placement on a chow diet **(E, F)**, at 14 weeks of age and four weeks after doxycycline removal and placement on a high-fat diet **(G, H),** and at 36 weeks of age and 26 weeks (6 months) after doxycycline removal and placement on a high-fat diet.

**Figure 6:**
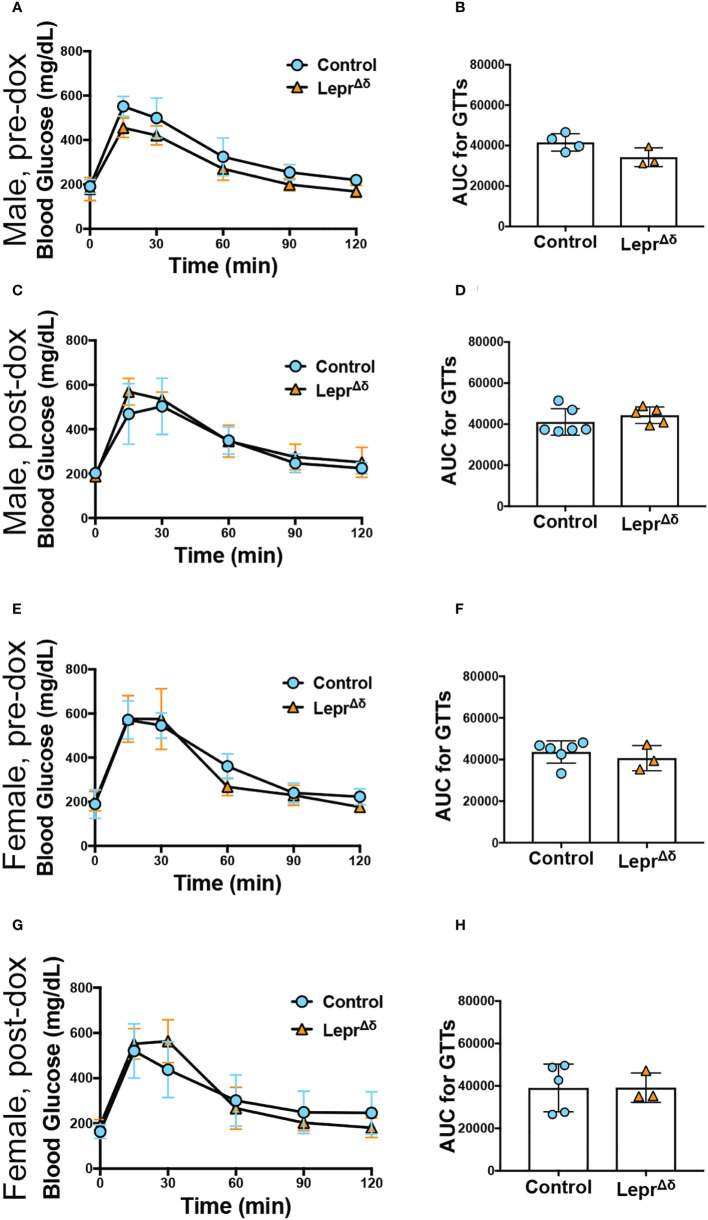
Male and female *Lepr^Δδ^* mice on a high-fat diet display normal glucose tolerance in response to a reduced glucose load. Glucose tolerance tests using 1g/kg glucose were performed before doxycycline treatment **(A, B, E, F)** and at 14 weeks of age in male **(C, D)** and female **(E-H)** *Lepr^Δδ^* and control mice on a HFD diet starting at 10 weeks of age. Glucose curves **(A, C, E, G)** and area-under-the-curve **(B, D, F, H)** are shown.

**Figure 7:**
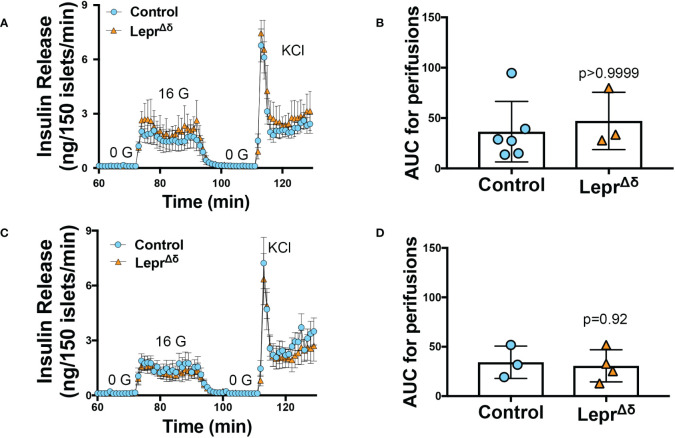
Islets from *Lepr^Δ^* mice display normal insulin secretion in response to high glucose. Isolated islets from 36-week old male **(A, B)** and female **(C, D)** *Lepr^Δδ^* and control mice were subjected to perifusions **(A, C)** at 0 mM glucose (0 G), 16 mM glucose (16 G), and with KCl. Both males **(A, B)** and females **(C, D)** were examined. Area-under-the-curve for 16 mM glucose is shown **(B, D)**.

Intraperitoneal GTTs were performed with 2g/kg glucose at 8 weeks, 14 weeks, and 36 weeks of age, corresponding to the pre-doxycycline time point, and then four weeks or six months after doxycycline removal. No difference was observed in glucose tolerance in male (**Figure 4**) or female (**Figure 5**) *Lepr^Δδ^* mice at any time point, whether the mice were on a standard chow or high-fat diet. To rule out the possibility that we were missing subtle differences, GTTs were also performed on males and females on a HFD diet using 1g/kg glucose (**Figure 6**). Again, no alterations in glucose tolerance were observed. Insulin secretion was examined by islet perifusion at 36 weeks of age. Islets from male and female *Lepr^Δδ^* mice displayed no significant differences in insulin secretion compared to controls (**Figures 7A-D**).

### Lepr is expressed in endothelial cell clusters within the mouse islet

**Figure 8:**
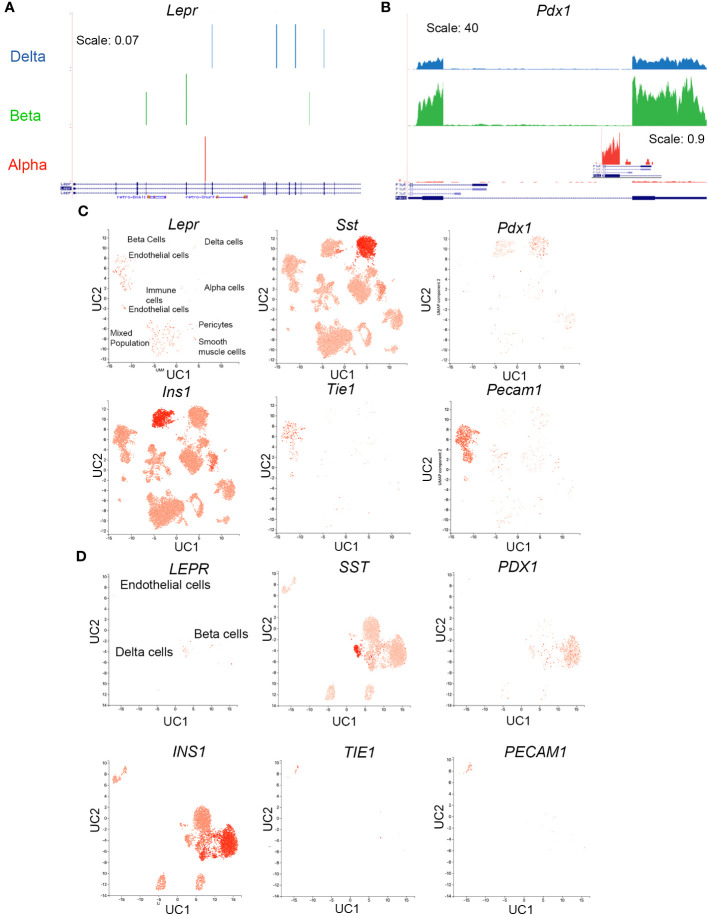
*Lepr* is not expressed in mouse delta cells. **(A, B)** RNAseq reads for *Lepr* **(A)** and *Pdx1* **(B)** in sorted alpha, beta, and delta cells were obtained from publicly available data. An inset for exon 1 *Pdx1* expression in alpha cells at a zoomed-in scale is included to demonstrate the appearance of lower-expressed genes. **(C)** scRNAseq expression in mouse islets cells for *Lepr*, *Sst*, *Pdx1, Ins*, and two endothelial markers, *Tie1* and *Pecam*, was obtained from the public database PanglaoDB (https://panglaodb.se SRA745567). **(D)** scRNAseq expression in human islet cells for *LEPR, SSST, PDX1, INS, TIE1*, and *PECAM* was obtained from PanglaoDB (SRA701877).

We next examined *Lepr* expression in mouse alpha, beta, and delta cells using a published dataset built from fluorescently sorted populations of live lineage-traced cells. *Lepr* was not detected in mouse alpha, beta, or delta cells (**Figure 8A**, (31)). This result starkly contrasts those for the transcription factor *Pdx1*, which is observed at the RNA level in each population (**Figure 8B**) despite the limitation of PDX1 protein expression to beta and delta cells.

Examination of a publicly available scRNAseq dataset uploaded to the PanglaoDB webserver with visualization capability (51) confirmed a lack of *Lepr* expression within the mouse islet endocrine population (**Figure 8C**). Most *Lepr*-positive cells are located in endothelial cell clusters (labeled in **Figure 8C**). An additional cell cluster exhibits *Lepr* expression; cells in this cluster expressed markers of several cell types, including *Tie-1*, *Sst*, *Ins*, *Pecam*, and *Pdx1*, among others. These cells may be doublets, the droplets capturing these cells may have been severely contaminated with RNA from lysed cells, or the number of genes sequenced in these cells may be too low for good segregation, since the mean number of genes per cell was 1528 (52, 53). This cluster contains many *Tie-1^+^* and *Pecam^+^* cells, both of which are endothelial cell markers.

Because islet *Lepr* expression appears within the mouse endothelial cell population, we assessed whether it is also expressed in human islet endothelial cells, again using publicly available data uploaded to Panglao DB (51). Unlike in the mouse islet, only a small percentage of cells within the human islet endothelial cluster expressed *LEPR* and most are observed in the delta-cell cluster (**Figure 8D**).

## Conclusions

Multiple labs have demonstrated that leptin decreases insulin secretion from isolated islets from multiple species. In addition, beta cells from control mice hyperpolarize in response to leptin, but this response is lost in beta cells from global *Lepr* null mice (54). These data suggest that a cell type within the islet mediates altered insulin secretion in response to leptin. However, pinpointing this cell type has proven to be challenging.

*Pdx1-Cre* is expressed throughout the pancreatic epithelium; this Cre line induces recombination of *Lepr* in the islet endocrine and exocrine cells but not in the immune, endothelial, or smooth muscle cells isolated along with the islet (55). *Pdx1-Cre;Lepr^fl/fl^* male and female mice on a normal chow diet exhibited improved glucose tolerance, normal insulin tolerance, and increased fasting serum insulin (18). Isolated *Pdx1-Cre;Lepr^fl/fl^* islets display increased Ca^2+^ influx and insulin secretion compared to *Lepr^fl/fl^* controls without Cre. Furthermore, leptin treatment represses insulin secretion in isolated islets from control but not *Pdx1-Cre;Lepr^fl/fl^* mice (18).

Many Cre lines that are expressed in the islet are also expressed in other tissues. *RIP-Cre* is expressed in the brain, including in hypothalamic neurons, and *RIP-Cre^25Mgn^* deletion of *Lepr* results in obesity, impaired glucose-stimulated insulin secretion, and a seemingly paradoxical reduced fasting blood glucose (19, 56). *Pdx1-Cre* is also expressed in the brain, primarily in the hypothalamus, and the overall *Pdx1-Cre;Lepr^fl/fl^* phenotype may result from this limited neuronal *Lepr* ablation (22, 56); however, whether neuronal deletion can explain altered insulin secretion Ca2^+^ influx in islets isolated from *Pdx1-Cre;Lepr^fl/fl^* mice is less clear. Neuronal effects on islets have been reported to be lost ex vivo (57).

Glucagon and *Gcg-Cre* are expressed in the hindbrain, olfactory neurons, and gut enteroendocrine L cells, as well as in islet alpha cells (21). The transgenic *Gcg-Cre* exhibits silencing, with recombination in only 30-45% of alpha cells, and the inducible gene replacement *Gcg-Cre^ERT2^* line suffers from glucagon haploinsufficiency (58). Somatostatin, *Sst-Cre*, and the *Sst-rtTA* used here are all expressed in gut enteroendocrine D cells, hypothalamic neurons, and the motor cortex (50).

Had the alpha or delta-cell *Lepr* ablation models resulted in altered glucose homeostasis, the cell type responsible would have been unclear. When using inducible *Sst-* and *Gcg-*driven lines, though, it is important to note a washout period can allow high-turnover cell populations, like gut enteroendocrine cells, to regenerate while the more slow-growing islet endocrine cells retain their deletion.

The *Pdx1-Cre;Lepr^fl/fl^* phenotype data suggest that pancreatic epithelial cells are responsible for the leptin-induced depression of glucose-stimulated insulin secretion in isolated islets. However, loss of *Lepr* in alpha cells using an inducible *Glucagon-Cre* (21), in beta cells using *Ins1-Cre* (21), or in delta cells (demonstrated here) does not result in altered glucose tolerance or insulin secretion. Additionally, bulk RNAseq of sorted mouse alpha, beta, or delta cells cannot detect *Lepr*. In addition, when we examined *Lepr* expression in control and *Lepr^Δδ^* islets, we saw no difference in *Lepr* expression. We initially attributed this lack of change despite seeing recombined DNA to the rarity of the delta cell. However, after examining *Lepr* in mouse islet scRNAseq data and in RNAseq data from sorted alpha, beta, and delta cells, we now deduce that detectable *Lepr* in the islet is likely expressed in endothelial cells (11, 18).

We unsuccessfully attempted to detect LEPR protein expression in the mouse pancreas using immunofluorescence and immunohistochemistry using an antibody previously used to detect LEPR mouse endothelial cells in other tissues (59). These data may indicate that no LEPR protein is synthesized in the pancreas; however, multiple groups have had difficulty immunolabeling LEPR, even in the hypothalamus (60). Because of the difficulty of LEPR immunolabeling, *Lepr-Cre* lines have been utilized in conjunction with a reporter mouse to detect *Lepr* expression patterns. In a *Lepr-Cre;TdTomato* line, TdTomato did not colocalize with insulin or glucagon in tissue sections, and the images shown had no TdTomato expression within the islets (61). However, TdTomato was detected in cells located in circular structures outside of the islets resembling blood vessels.

*Lepr* is expressed in mouse T cells (62, 63), endothelial cells (64), and smooth muscle cells (64). Here, we show the presence of *Lepr* in mouse islet endothelial cells using publicly available RNAseq data. These data suggest that, in the mouse, islet endothelial cells mediate the effect of leptin on insulin secretion. However, mice with *Lepr* deleted specifically in endothelial cells using a *Tie2-Cre* display unchanged *ad libitum*-fed blood glucose (41, 59). A more systematic analysis of glucose homeostasis was not performed.

Endothelial cells regulate beta-cell function and insulin release through factors such as Endothelin-1 and Thrombospondin-1 (65, 66). Thus, signaling changes between endothelial and beta cells in response to leptin treatment may mediate decreased insulin secretion in mouse islets. However, *Pdx1-Cre* should not have activity in endothelial cells; therefore, the endothelial cells are unlikely to underlie altered insulin secretion in the *Pdx1-Cre;Lepr^fl/fl^* model. In human islets, *LEPR* is seen in scant endothelial cells by scRNAseq; thus, in human islets, any effect of leptin on insulin release is unlikely through endothelial cells. Instead, *LEPR* enrichment within the human delta-cell population suggests that the delta cell mediates the reduced insulin secretion response to leptin in human islets.

In contrast to what likely occurs in humans, we provide evidence that the mouse delta cell does not control this response. Further work will be necessary to definitively determine how leptin affects insulin secretion in various species. These data should serve as a deterrent from extrapolating the effects and mechanisms of islet leptin treatment from one species to another.

## Data availability statement

Original datasets are available in a publicly accessible repository: https://www.ncbi.nlm.nih.gov/geo/, accession number: GSE243984. Publicly available datasets were analyzed in this study and can be found at hpap.pmacs.upenn.edu. Publicly available datasets were used as previously analyzed and can be found at https://huisinglab.com/islet_txomes_2016/index.html and at https://panglaodb.se/, accession numbers SRA745567 and SRA701877. All other data is available upon reasonable request.

## Ethics statement

For studies involving human cadaveric material, a waiver was obtained by Johns Hopkins Institutional Review Board. The studies were conducted in accordance with local legislation and institutional requirements. The human samples used in this study were acquired from the Integrated Islet Distribution Program, funded by Special Statutory Funding Program for Type 1 Diabetes Research, administered by the NIDDK. The IIDP works with islet isolation centers throughout the United States to provide de-identified islets from cadaveric donors to researchers. Written informed consent for participation was obtained from relatives of donors by OPOs before the IIDP received and disseminated islets. The animal studies were approved by Johns Hopkins University Institutional Animal Care and Use Committee. The studies were conducted in accordance with the local legislation and institutional requirements.

## Author contributions

JZ: Conceptualization, Formal Analysis, Investigation, Writing – review & editing. KK: Formal Analysis, Investigation, Writing – review & editing. EM: Formal Analysis, Investigation, Writing – review & editing. AY: Formal Analysis, Investigation, Writing – review & editing. GP: Investigation, Writing – review & editing. MG: Conceptualization, Formal Analysis, Funding acquisition, Investigation, Methodology, Resources, Supervision, Writing – original draft, Writing – review & editing.
